# Hyperexcitability of Sensory Neurons in Fragile X Mouse Model

**DOI:** 10.3389/fnmol.2021.796053

**Published:** 2021-12-22

**Authors:** Pan-Yue Deng, Oshri Avraham, Valeria Cavalli, Vitaly A. Klyachko

**Affiliations:** ^1^Department of Cell Biology and Physiology, Washington University School of Medicine, St. Louis, MO, United States; ^2^Department of Neuroscience, Washington University School of Medicine, St. Louis, MO, United States; ^3^Hope Center for Neurological Disorders, Washington University School of Medicine, St. Louis, MO, United States; ^4^Center of Regenerative Medicine, Washington University School of Medicine, St. Louis, MO, United States

**Keywords:** hyperexcitability, Fragile X syndrome, action potential, sensory neuron, HCN channel

## Abstract

Sensory hypersensitivity and somatosensory deficits represent the core symptoms of Fragile X syndrome (FXS). These alterations are believed to arise from changes in cortical sensory processing, while potential deficits in the function of peripheral sensory neurons residing in dorsal root ganglia remain unexplored. We found that peripheral sensory neurons exhibit pronounced hyperexcitability in *Fmr1* KO mice, manifested by markedly increased action potential (AP) firing rate and decreased threshold. Unlike excitability changes found in many central neurons, no significant changes were observed in AP rising and falling time, peak potential, amplitude, or duration. Sensory neuron hyperexcitability was caused primarily by increased input resistance, without changes in cell capacitance or resting membrane potential. Analyses of the underlying mechanisms revealed reduced activity of HCN channels and reduced expression of HCN1 and HCN4 in *Fmr1* KO compared to WT. A selective HCN channel blocker abolished differences in all measures of sensory neuron excitability between WT and *Fmr1* KO neurons. These results reveal a hyperexcitable state of peripheral sensory neurons in *Fmr1* KO mice caused by dysfunction of HCN channels. In addition to the intrinsic neuronal dysfunction, the accompanying paper examines deficits in sensory neuron association/communication with their enveloping satellite glial cells, suggesting contributions from both neuronal intrinsic and extrinsic mechanisms to sensory dysfunction in the FXS mouse model.

## Introduction

Fragile X syndrome (FXS) is the leading monogenetic cause of intellectual disability (ID) and autism. This disorder most commonly results from transcriptional silencing of the *Fmr1* gene causing loss of expression of Fragile X Mental Retardation Protein (FMRP) (Penagarikano et al., 2007). Individuals with FXS typically present with cognitive dysfunction, learning deficits, social and behavioral problems, neurological deficits, and morphological abnormalities. Among most prevalent FXS deficits is hypersensitivity to sensory stimuli, including auditory, visual, and tactile stimuli. Increasing evidence suggests that sensory hypersensitivity may lead to behavioral alterations such as poor eye contact, anxiety, and impaired social interactions (Rais et al., 2018).

Individuals with FXS commonly exhibit somatosensory deficits, such as hypersensitivity to touch (Cascio, 2010), Self-injurious behaviors in Fragile X individuals (Arron et al., 2011; Crawford et al., 2019) are also indicative of abnormal pain perception. Impaired pain induction and perception are also observed in the FXS mouse model, including reduced induction of neuropathic pain (Ramirez-Lopez et al., 2021), and insensitivity to visceral pain (Yang et al., 2020). These and other sensory deficits have been largely attributed to alterations in cortical sensory processing with a wide range of excitability deficits identified in somatosensory cortex of FXS models at neuronal, circuit, and network levels (Contractor et al., 2015). For example, *Fmr1* KO mice have abnormal encoding of tactile stimulation frequency and enlarged receptive fields in the somatosensory cortex (Juczewski et al., 2016). However, recent studies in other monogenetic models of autism suggest that many core cognitive and sensory deficits may arise from an earlier abnormality in sensory inputs that drive subsequent abnormal development of cortical circuits (Orefice et al., 2016, 2019). A hyperexcitable state of somatosensory neurons has been suggested to be a part of the core developmental pathology in autism models, leading to region-specific brain abnormalities during the critical period (Orefice et al., 2019). Indeed, the formation of the cortical receptive fields depends on sensory experience (Allen et al., 2003). The enlarged receptive fields in *Fmr1* KO mice and altered perception of sensory information may be a consequence, in part, of altered sensory inputs during development. Yet little is known about alterations in the peripheral neural system that receives the primary sensory inputs. Research in Fragile X field has almost exclusively focused on central defects in processing of somatosensory information and dysfunction of the central neurons and circuits. Whether dysfunction of peripheral sensory neurons is present and contributes to FXS pathophysiology remains largely unexplored.

Peripheral sensory neurons in the dorsal root ganglia (DRG) play critical roles in receiving direct sensory information from the environment and conveying it to the central nervous system (CNS). Structurally, these are pseudo-unipolar neurons with one axon projecting into peripheral nerve and the other axon ascending in the dorsal root and spinal cord. Sensory neurons express FMRP, which localize to the soma and axons (Price et al., 2006). While the gross development of DRG is normal in the absence of FMRP (Price and Melemedjian, 2012), there is evidence for functional defects in sensory neurons including an increased surface expression of voltage-gated calcium channels leading to increased somatic glutamate release (Ferron et al., 2014, 2020). However, whether and how excitability of peripheral sensory neurons is altered by the loss of FMRP remains unexplored.

In this study we performed recordings from the DRG neurons isolated from adolescent mice in a short-term culture to examine changes in sensory neuron excitability caused by loss of FMRP.

## Results

### Hyperexcitable State of Sensory Neurons in *Fmr1* KO Mice

**FIGURE 1:**
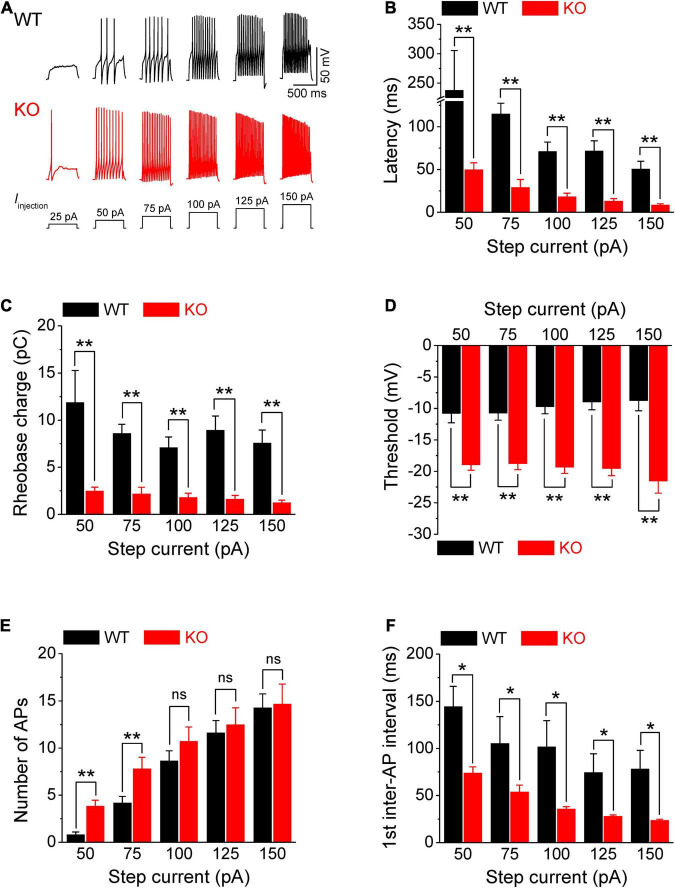
Increased excitability of sensory neurons in *Fmr1* KO mice. **(A)** Sample AP traces evoked by step-current injection. Note, that for a 25-pA step, there was no AP observed in any of tested WT neurons, and in ∼50% of KO neurons. Thus, in the following panels we analyzed AP parameters starting from 50 pA step. **(B–F)** Analysis of AP parameters in measurements from panel **(A)**. Latency to the first AP **(B)**, rheobase charge transfer **(C)**, threshold **(D)**, number of AP fired **(E)**, and first inter-AP interval **(F)** in WT and *Fmr1* KO neurons. *T*-test; *p < 0.05; **p < 0.01; ns, not significant.

Firing patterns of sensory neurons situated in the DRG convey information from external and internal environment of the body to the CNS. Thus, these neurons play a critical role in transducing sensory information to neuronal signals. Accordingly, we first asked how excitability of sensory neurons is affected in *Fmr1* KO mice. Whole-cell recordings were performed in short-term cultures of sensory neurons, as described (Avraham et al., 2020). Neurons were separated for analysis by size into two groups with a cutoff at 30 μm diameter (Yousuf et al., 2019) into small/medium vs. large diameter neurons. We found that most of the small/medium diameter neurons [mean diameter: 19.14 ± 0.53 μm (WT), 19.84 ± 0.54 μm (KO)] in the short-term DRG cultures exhibit tonic action potential (AP) firing (multiple APs fired in a stimulus intensity-dependent manner, Figure 1A), whereas all tested large neurons (>30 μm) show phasic AP firing (a single AP fired regardless of stimulus intensity, data not shown). To better understand how and to what extent the excitability of somatosensory neurons is affected in *Fmr1* KO mice, we therefore used the small/medium diameter sensory neurons as a model neuron. In our culture conditions, the majority of the cells we recorded from are small/medium diameter IB4-positive neurons (Avraham et al., 2020). IB4 positive neurons represent non-peptidergic C- and Aδ-nociceptive neurons and some Aδ-low threshold mechanoreceptors (Wang et al., 1998; Li et al., 2011; Olson et al., 2016), a subset of which detect gentle touch (Liu et al., 2007; Olson et al., 2016).

**FIGURE 2:**
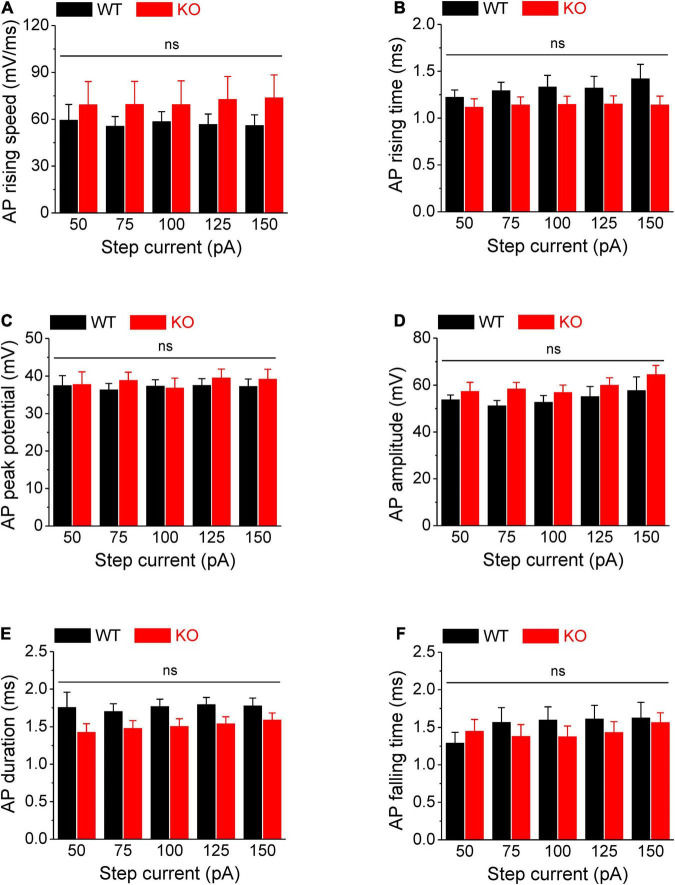
No changes in AP rising and falling time, duration, and amplitude in *Fmr1* KO neurons. **(A–F)** Loss of FMRP did not affect AP rising speed **(A)**, 10–90% rising time **(B)**, AP peak potential **(C)**, AP amplitude **(D)**, AP duration **(E)**, and 90–10% AP falling time **(F)**. *T*-test; ns, not significant.

Action potentials were evoked by multi-step current injections (from 25 to 150 pA with a step size of 25 pA, Figure 1A). Only the first APs were used to determine AP latency, threshold, and rheobase to avoid AP parameters being affected by cumulative Na^+^ channel inactivation during subsequent APs. We found that excitability of sensory neurons was markedly increased in the absence of FMRP, as evident by decreased latency to the first AP (*p* < 0.01, Figure 1B, all values here and throughout are summarized in Supplementary Table 1), lower voltage threshold (*p* < 0.01, Figure 1D), and reduced rheobase charge transfer (*p* < 0.01, Figure 1C) which represents a measure of current threshold. Sensory neurons in *Fmr1* KO mice also fired more APs at lower stimulus intensity steps (*p* < 0.01, Figure 1E), and had a shortened inter-AP interval (*p* < 0.05, Figure 1F). Unlike excitability changes observed in many central neurons (Contractor et al., 2015) we observed no significant changes in AP rising and falling time, AP peak potential, and AP amplitude, as well as in AP duration (Figure 2). It is noteworthy that the threshold and rheobase values were largely independent of step current intensities within genotypes, indicating that these are reliable parameters for evaluating neuronal excitability.

**FIGURE 3:**
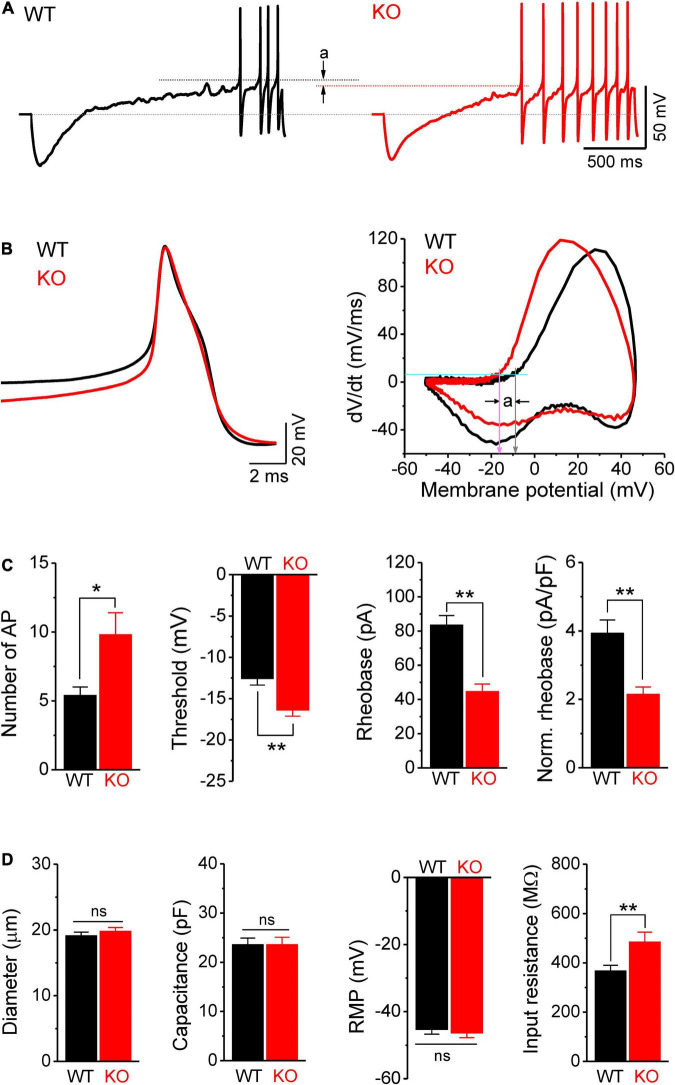
Hyperexcitability of sensory neurons in *Fmr1* KO mice is associated with increased input resistance. **(A)** Sample traces of ramp-evoked APs. Note the differences in number of APs and threshold (a) between WT and KO neurons. Short dot-lines indicate the threshold levels of WT (black) and KO (red) neurons. Long dot-line is the resting membrane potential (RMP) level. **(B)** The first APs from panel **(A)** and their corresponding phase plots, indicating decreased AP threshold in *Fmr1* KO mice (a). Cyan line is the membrane depolarizing speed of 5 mV/ms, the corresponding voltages of its intersections with phase plot were thresholds for WT (gray arrow) and KO (pink arrow) neurons. **(C)** Summarized data for the number of APs, threshold, rheobase, and membrane capacitance-normalized rheobase for the measurements in panel **(A)**. **(D)** Summarized data for cell size, membrane capacitance, RMP, and membrane input resistance for the measurements in panel **(A)**. *T*-test; *p < 0.05; **p < 0.01; ns, not significant.

We further confirmed the increased excitability of sensory neurons in *Fmr1* KO mice by examining AP parameters in a ramp-current evoked AP traces, using a previously reported approach (Deng and Klyachko, 2016a,b; Deng et al., 2019). As expected, we found that excitability of DRG neurons was indeed increased in *Fmr1* KO mice, as evident by a significantly larger number of APs fired in KO neurons (*p* = 0.014; Figures 3A,C); a large hyperpolarizing shift of threshold potential (*p* = 0.0015; Figures 3A–C), and reduced AP rheobase (*p* < 0.0001; Figure 3C).

Together, these observations demonstrate a state of marked hyperexcitability of peripheral sensory neurons in the absence of FMRP.

### Hyperexcitability of Sensory Neurons in *Fmr1* KO Mice Is Associated With Increased Input Resistance, but Is Not Due to Kv7 Channel Deficits

Because the intrinsic membrane properties play a major role in setting neuronal excitability, we examined the resting membrane potential (RMP), cell size, membrane capacitance, and input resistance of sensory neurons in *Fmr1* KO and WT animals. While no significant differences were observed in cell size, capacitance, and RMP between genotypes (Figure 3D), we found a significant increase in input resistance in *Fmr1* KO neurons (*p* = 0.0097; Figure 3D). Increased input resistance is consistent with the reduced rheobase in the absence of FMRP and may thus be a direct cause of hyperexcitability of KO neurons.

To understand the mechanisms of these excitability defects, we first considered that absence of significant changes in RMP or AP waveform suggests that the voltage-gated Na^+^ and K^+^ channels active near RMP or above threshold are unlikely to be strongly affected in sensory neurons of *Fmr1* KO mice. Thus, the changes in input resistance may result from alterations in some voltage-dependent sub-threshold conductance, such as M current (carried by Kv7 channels) and/or h current (carried by HCN channels) that are abundantly expressed in sensory neurons (de Moraes et al., 2017). We examined contributions from Kv7 channels and found that the Kv7 channel blocker XE991 (10 μM) failed to abolish the differences between genotypes in either the voltage threshold (*p* = 0.012, Supplementary Figure 1A), or in the rheobase charge transfer (*p* < 0.0001; Supplementary Figure 1B). This observation suggests that Kv7 channels are unlikely to underlie excitability changes observed in *Fmr1* KO neurons.

### HCN Channel Dysfunction Causes Hyperexcitability of Sensory Neurons in *Fmr1* KO Mice

**FIGURE 4:**
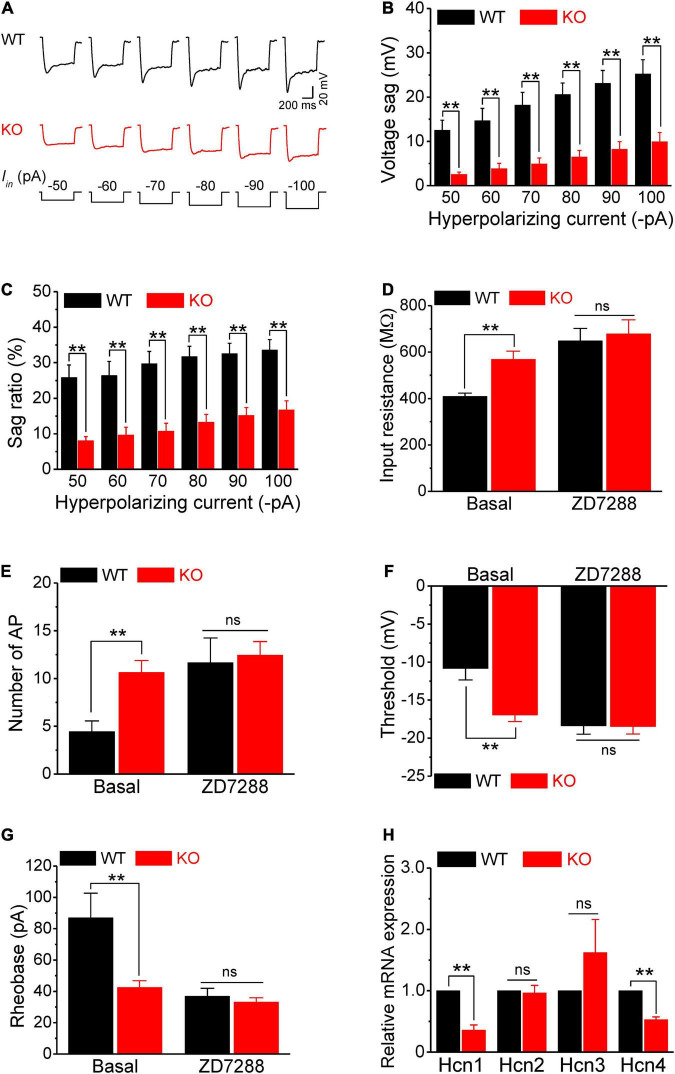
HCN channel dysfunction causes hyperexcitability of sensory neurons in *Fmr1* KO mice. **(A)** Example traces of hyperpolarization-induced voltage sag in sensory neurons. *I*_*in*_ is the injected hyperpolarizing current with intensities indicated in the lower panel. **(B,C)** Quantification of voltage sag in WT and KO neurons in absolute values **(B)** and as a sag ratio **(C)** for all tested hyperpolarizing current levels. **(D)** A selective HCN channel blocker ZD7288 abolished the differences in input resistance between genotypes. **(E–G)** HCN channel blocker ZD7288 abolished the differences in the number of APs fired **(E)**, threshold **(F)**, and rheobase **(G)** between genotypes. **(H)** qPCR analysis of the relative mRNA expression of *Hcn1–4* in *Fmr1* KO compared to WT DRGs. *N* = 3 biologically independent animals. *T*-test; **p < 0.01; ns, not significant.

Next, we examined the HCN channel activity in sensory neurons. HCN channels are activated by a membrane hyperpolarization, and cells with active HCN channels are characterized by voltage sag in current clamp recordings in response to a hyperpolarizing current. As expected, negative current injection produced marked voltage sags in WT neurons (Figure 4A). Most importantly, the voltage sag in *Fmr1* KO neurons was significantly decreased compared to WT in all tested steps for both absolute values and sag ratios (*p* < 0.01 for all steps; Figures 4A–C). These differences were not due to the basal RMP differences between genotypes (*p* = 0.52, Supplementary Table 1).

Given that the HCN channels contribute significantly to input resistance, these results suggest that the reduced HCN channel function might be the major cause of hyperexcitability in sensory neurons of *Fmr1* KO mice. If this is the case, inhibition of HCN channels should eliminate the differences in input resistance and in excitability of sensory neurons between genotypes. In line with this prediction, the HCN channel blocker ZD7288 (10 μM), which potently blocks all HCN channels without preference for a specific HCN subunit, abolished the difference in input resistance between WT and KO neurons (*p* = 0.73; Figure 4D). Most importantly, ZD7288 also abolished differences in sensory neuron excitability between WT and KO neurons, including the number of AP fired (*p* = 0.78; Figure 4E), AP threshold (*p* = 0.94; Figure 4F), as well as rheobase (*p* = 0.50; Figures 4G,H). Together, these results confirmed that reduced HCN channel function causes increased input resistance, which in turn leads to hyperexcitability of sensory neurons in *Fmr1* KO mice.

Fragile X mental retardation protein regulates expression and activity of multiple ion channels (Deng and Klyachko, 2021) and HCN channels are a known target of FMRP translational control (Darnell et al., 2011; Brager et al., 2012; Zhang et al., 2014; Orefice et al., 2016, 2019). We thus examined by quantitative PCR (qPCR) if *Hcn* channel expression was altered in sensory neurons of *Fmr1* KO mice. We used whole DRG for these experiments, since HCN channels are selectively expressed in neurons in the DRG (Moosmang et al., 2001; Biel et al., 2009), with HCN1, HCN2, and to a lesser extent HCN4 being most abundant (Moosmang et al., 2001; Chaplan et al., 2003). We found that mRNA levels of *Hcn1* and *Hcn4* were significantly reduced in *Fmr1* KO DRG (*Hcn1*, *p* = 0.0018; *Hcn4*, *p* = 0.0004) (Figure 4H) while levels of *Hcn2* and *Hcn3* were not strongly affected (*Hcn2*, *p* = 0.78; *Hcn3*, *p* = 0.32) (Figure 4H). Together with the above results, this observation suggests that neuronal hyperexcitability in the absence of FMRP is caused by reduced HCN channel expression in sensory neurons.

## Discussion

Sensory hypersensitivity in FXS has thus far been largely attributed to sensory processing abnormalities in the brain circuits (Contractor et al., 2015). Our results revealed a contribution to sensory abnormalities from peripheral deficits in the FXS mouse model. We found a hyperexcitable state of peripheral sensory neurons characterized by markedly increased AP frequency and reduced threshold caused by loss of FMRP. Altered sensory neuron excitability in *Fmr1* KO mice arises, at least in part, from intrinsic neuronal mechanisms involving increased input resistance caused by HCN channel dysfunction.

HCN channels are active at rest and play a crucial role in controlling input resistance, and thus neuronal excitability (Shah, 2014). Voltage dependence of HCN channels is regulated by a number of intracellular factors. Other voltage-gated channels have a strong influence on HCN channel activity. The resulting action of HCN channels on membrane excitability in a given cell type is thus determined by a delicate balance of these factors. For example, in the CA1 neurons, through interaction with Kv7 channels, HCN channels can enhance AP firing in response to an EPSP when AP threshold is low and can inhibit AP firing when AP threshold is high (George et al., 2009). The AP threshold of DRG neurons is comparatively high, thus reduction of HCN channel activity in *Fmr1* KO neurons is consistent with increased firing. Also, HCN channels have two separate influences on membrane excitability: one is the channel-mediated inward current (i.e., excitatory influence), the second one is a shunting effect (inhibitory influence). The overall effect might be complicated or even “paradoxical” (George et al., 2009). Given that the input resistance is a direct determinant of AP rheobase, our observations of reduced rheobase in *Fmr1* KO neurons support the notion that HCN channels in sensory neurons function through the shunting effect to reduce input resistance.

Cell-type specific HCN channel defects have been previously implicated in excitability alterations of central neurons in the FXS models. For example, the elevated HCN1 subunit expression and increased Ih current were found in dendrites of CA1 pyramidal cells from *Fmr1* KO mice leading to decreased input resistance and reduced temporal summation (Brager et al., 2012), while the opposite changes in HCN1 expression, Ih and excitability were observed in the layer 5 pyramidal cells of somatosensory cortex (Zhang et al., 2014) or layer 4 stellate cells of *Fmr1* KO mice (Booker et al., 2019). Reduced HCN1 expression and decreased Ih were also found in large (mechanosensory) but not small diameter DRG neurons in Shank3 deletion model of Phelan–McDermid syndrome often associated with ASD (Orefice et al., 2016, 2019). Here we observed that *Hcn1* and *Hcn4* expression is strongly reduced in the DRG of *Fmr1* KO mice. Together with the observations that a selective HCN channel blocker abolished differences in all measures of excitability between *Fmr1* KO and WT mice, these observations suggest that reduced HCN channel expression is the major cause of hyperexcitability of sensory DRG neurons caused by FMRP loss. Notably, in central neurons, FMRP is also known to exert powerful control over ion channel activity *via* protein–protein interactions (Brown et al., 2010; Deng et al., 2013, 2019; Deng and Klyachko, 2021). In the case of HCN channels, FMRP can regulate the channel’s surface levels in a tissue-specific manner *via* protein–protein interactions with the HCN-TRIP8b complexes (Brandalise et al., 2020). Whether this interaction is present in sensory neurons and contribute to neuronal excitability defects remains to be determined.

Interestingly, HCN channel expression and Ih current show age-dependent increase in thalamic neurons (Kanyshkova et al., 2009) which dampens their excitability as these neurons mature. Furthermore, there is evidence of maturation-dependent regulation of HCN channels in spiral ganglia in the auditory pathway (Shen et al., 2018) and immature neurons are known to exhibit higher intrinsic excitability and plasticity (Schmidt-Hieber et al., 2004; Oh et al., 2010). Can hyperexcitability of peripheral sensory neurons be a consequence, in part, of the delayed neuronal maturation in *Fmr1* KO mice? The age-dependent changes in excitability have been reported in central neurons of *Fmr1* KO mice: the CA3 pyramidal neurons show increased excitability in young *Fmr1* KO mice (3–4 weeks) (Deng et al., 2019; Dwivedi et al., 2019), but this was not seen in the older animals (6–8 weeks) (Dwivedi et al., 2019). Further, a delay in neuronal maturation and immature state of dendritic spines is widely documented in central neurons of *Fmr1* KO mice (Comery et al., 1997; Harlow et al., 2010; Guo et al., 2015; Moskalyuk et al., 2020), resulting in delayed maturation of local networks (Vislay et al., 2013; Nomura et al., 2017) and a developmental delay in somatosensory map formation (Till et al., 2012). This is also consistent with abnormal neurogenesis and altered differentiation of neural stem cells in *Fmr1* KOs, leading to poor neuronal maturation and high gliogenic development (Castren et al., 2005; Telias et al., 2013, 2015). Our single-cell RNA-seq analyses described in detail in the accompanying paper indeed suggest that maturation of sensory neurons in the DRG is delayed/aberrant, as evident in upregulation of progenitor markers and downregulation of neuronal differentiation/neuronal identity markers. Thus, the delayed maturation of sensory neurons in the DRG could be an underlying or contributing factor driving their hyperexcitability.

The increased intrinsic excitability of sensory neurons is only one of complex multifaceted changes that occur in the peripheral sensory system in the absence of FMRP. For example, the excitability changes we observed here will combine with the increased glutamate release from the soma and terminals of these neurons, which occurs independently due to excessive surface expression of N-type calcium channels (Ferron et al., 2014, 2020), further multiplying the excessive output from sensory neurons. Moreover, morphological changes of neuronal processes, such as axon structure or axon initial segment length, which are affected centrally in *Fmr1* KO mice (Booker et al., 2020), can also contribute to sensory neuron excitability. Given the long-range projections of the sensory neurons of the DRG, future central and peripheral projection tracing will be needed to define the precise morphological changes in sensory neuron processes. In addition to these intrinsic mechanisms, in the accompanying paper, we describe extrinsic mechanisms that may contribute to sensory deficits due to disruption of the peripheral neurons’ association/communication with their enveloping satellite glial cells. Thus, an interplay of multiple peripheral deficits needs to be considered to fully understand sensory deficits caused by FMRP loss. Notably, our measurements of neuronal excitability are limited to short-term cultures in which neurons do not develop full length long-range projections and do not get enveloped by the satellite glia cells. Thus how the complex interplay between the intrinsic and extrinsic changes influences sensory processing *in vivo* remains to be elucidated. This includes defining how sensory transduction is affected in the intact DRG and whether distorted cortical maps in *Fmr1* KOs (Till et al., 2012) are a consequence of altered sensory receptive fields. Moreover, *in vivo* measurements will also be needed to define the extent to which peripheral deficits contribute to the abnormal processing of repeating sensory stimuli (Domanski et al., 2019). Such measurements will present a technical challenge because sensory DRG neurons in more intact settings (*ex vivo* slices or *in vivo*) are entirely surrounded by the satellite glia coat.

What is the relevance of sensory neuron hyperexcitability to clinical FXS phenotypes? The majority of cells analyzed in our experiments were small/medium diameter IB4-positive nociceptors (Avraham et al., 2020). A previous study showed that loss of FMRP decreases nociceptive sensitization in adult mice, even though the basal nociceptive thresholds were intact (Price et al., 2007). Recent evidence also indicates impaired pain induction and perception in the FXS mouse model, including reduced neuropathic pain (Ramirez-Lopez et al., 2021), and visceral pain (Yang et al., 2020). Future *in vivo* studies will be needed to determine whether and how increased excitability of nociceptive neurons in adolescent mice we observed here is linked to abnormal pain induction or perception in FXS mice. Notably, IB4-positive sensory neurons also include a subset of mechanoreceptors that detect gentle touch (Liu et al., 2007). Thus, our observations could be relevant to the clinical FXS phenotypes beyond the pain induction/perception, since individuals with Fragile X are known to experience hypersensitivity to touch (Arnett et al., 2014; He et al., 2017).

## Materials and Methods

### Animals and Dorsal Root Ganglia Neuronal Culture

*Fmr1* KO (FVB.129P2-Pde6b^+^Tyr*^c–ch^*Fmr1*^tm1Cgr^*/J; stock #004624) and WT control (FVB.129P2-Pde6b^+^Tyr*^c–ch^*/AntJ; stock #004828) mice on FVB background were obtained from The Jackson Laboratory. Male mice (28- to 30-day old) were used for DRG cultures since male FXS individuals typically have more severe symptoms than do female individuals (Hagerman et al., 2009). Lumbar DRG (L1–L5) were dissected from *Fmr1* KO and WT control mice and collected into cold Hank’s balanced salt solution (HBSS) with 5% Hepes, then transferred to warm papain solution and incubated for 20 min in 37°C. DRG’s were washed in HBSS and incubated with collagenase for 20 min in 37°C. Ganglia were then mechanically dissociated to a single cell suspension by triturating in culture medium (Neurobasal medium), with Glutamax, PenStrep, and B-27. Cells were then cultured on 100 μg/ml poly-D-lysine coated cover slips and used for electrophysiological recording 24 h after plating. All animal procedures were in compliance with the NIH Guide for the Care and Use of Laboratory Animals and conformed to Washington University Animal Studies Committee guidelines.

### Electrophysiology

#### Action Potential Recording and Analysis

Whole-cell patch-clamp recordings in a current-clamp mode were performed using a MultiClamp 700B amplifier (Molecular Devices) from short-term cultures (24 h after plating) of isolated DRG neurons, visually identified with infrared video microscopy and differential interference contrast optics (Olympus BX51WI). Current-clamp recordings were made with pipette capacitance compensation and bridge-balance compensation. Recordings were conducted at near-physiological temperature (33–34°C). In these conditions, the majority of cells analyzed were small/medium diameter IB4-positive neurons (Avraham et al., 2020). The recording electrodes were filled with the following (in mM): 130 K-gluconate, 10 KCl, 0.1 EGTA, 2 MgCl_2_, 2 ATPNa_2_, 0.4 GTPNa, and 10 HEPES, pH 7.3. The extracellular solution contained (in mM): 145 NaCl, 3 KCl, 10 HEPES, 2.5 CaCl_2_, 1.2 MgCl_2_, and 7 glucose, pH 7.4 (saturated with 95% O_2_ and 5% CO_2_). APs were evoked either by multiple-step-current injection (from 25 to 150 pA with step duration of 600 ms and step size 25 pA) or by a ramp-current injection (0.1 pA/ms) with a hyperpolarizing onset. To determine the number of APs, all APs for each step were counted (step-evoked APs), but for the ramp-evoked APs, only APs within the first 2 s from beginning of the ramp were counted. AP threshold was defined as voltage where the AP rise speed reaches 5 mV/ms. The AP threshold was determined only from the first APs in the trace. For ramp-evoked APs, AP rheobase was determined as current amplitude difference from baseline to threshold point. Rheobase charge transfer was the integration of the current over the time interval, which was from the beginning of the steps (or ramp cross baseline) to the first AP threshold point. AP latency was defined as the time duration from the beginning of step-current to the first AP threshold point. AP duration was defined as the time interval between AP rising and falling parts at a membrane potential of +15 mV level. When the number of APs was more than 2, the first inter-AP interval was defined as the time duration between the peaks of first and second APs. All data were averaged over 5–8 trials for each cell. All chemicals for internal solution and bath solution were from Sigma-Aldrich. The channel blockers ZD7288 and XE991 were from Tocris. Different cells were used to test the effect of blockers (ZD7288 or XE991) to minimize the influence from “washout effect” due to recording time limitations (recordings in DRG cultures have a fast rundown during whole cell recordings).

#### Determination of Resting Membrane Potential, Capacitance, and Input Resistance

Resting membrane potential was measured immediately after whole-cell formation. Cell capacitance was determined by the amplifier’s auto whole-cell compensation function with slight manual adjustment to optimize the measurement if needed. Under current-clamp mode, a negative current (−50 pA for 500 ms) was injected every 5 s to assess the input resistance. The voltage difference between baseline and steady state was used to calculate input resistance.

#### Measurements of HCN Channel Activity

For evaluation of HCN channel activity, hyperpolarization-evoked voltage sag was determined by step-current injection (from −50 to −100 pA with step size −10 pA and duration 600 ms). Sag amplitude was defined as the voltage difference between the lowest point of voltage trace and steady-state part (average 50 ms) immediate before the end of step. Sag ratio was calculated as 100% × (sag amplitude) ÷ (voltage difference between baseline and the lowest point of voltage trace).

### RNA Isolation and Quantitative PCR

Dorsal root ganglia were lysed and total RNA was extracted using Trizol reagent (Thermo Fisher, Cat# 15596026). Next, RNA concentration was determined using a NanoDrop 2000 (Thermo Fisher Scientific). First strand synthesis was then performed using the High Capacity cDNA Reverse Transcription kit (Applied Biosystems). qPCR was performed using PowerUp SYBR Green master mix (Thermo Fisher, Cat# a25742) using 5 ng of cDNA per reaction. Plates were run on a QuantStudio 6 Flex system. Quantification of relative gene expression was performed using an automated software package (QuantStudio, ThermoFisher Scientific) following a standard 2^–ΔΔ^*^Ct^* method as described (Rao et al., 2013). Briefly, the cycle threshold (Ct) information generated by the qPCR system is directly used to determine relative gene expression in target and reference samples, using a reference gene as the normalizing factor (Rao et al., 2013). The Ct for the mRNA of a housekeeping gene (*Rpl13a*) was first subtracted from the Ct for the mRNA of the different *Hcn* isoforms in the same sample to normalize for variation in the amount and quality of mRNA between different samples. This normalization procedure (Δ*Ct*) permits comparison of expression of a gene of interest among different samples. The average Δ*Ct* value from three technical replicates was calculated for each of the biological replicates (*n* = 3). The final outcome of this quantification was calculated as the fold change of *Hcn* isoforms expression in the KO samples relative to their expression in the WT samples (ΔΔ*Ct*). The relative gene expression is usually set to 1 for reference samples (WT) because ΔΔ*Ct* is equal to 0 and therefore 2^0^ is equal to 1 (Rao et al., 2013).

Primer sequences were obtained from PrimerBank or published literature and product size validated using agarose gel electrophoresis.

Rpl13a (PrimerBank ID 334688867c2) Forward Primer AGCCTACCAGAAAGTTTGCTTAC Reverse Primer GCTTCTTCTTCCGATAGTGCATC.

Hcn1 Forward Primer ACATGCTGTGCATTGGTTATGGCG, Reverse PrimerAACAAACATTGCGTAGCAGGTGGC.

Hcn2 Forward Primer ACTTCCGCACCGGCATTGTTATTG, Reverse Primer TCGATTCCCTTCTCCACTATG AGG.

Hcn3 Forward Primer TGGGAACCACTGGTGCACG, Reverse Primer TGAGCGTCTAGCAGATCGAG.

Hcn4 Forward Primer GCATGATGCTTCTGCTGTGTCACT, Reverse Primer TTCACCATGCCATTGATGGACACC.

### Statistical Analysis

Data are presented as mean ± SEM. Student’s *T*-test was used for statistical analysis as appropriate. Significance was set as *p* < 0.05. The *n* was number of cells tested. All statistical values and tests used in each experiment are given in Supplementary Table 1 for each panel.

## Data Availability Statement

The raw data supporting the conclusions of this article will be made available by the authors, without undue reservation.

## Ethics Statement

All animal procedures were reviewed and approved by the Washington University School of Medicine Institutional Animal Care and Use Committee (IACUC) under protocol A-3381-01. All experiments were performed in accordance with the relevant guidelines and regulations. All experimental protocols involving mice were approved by the Washington University School of Medicine (protocol #21-0104 and #20-0173). Mice were housed and cared for in the Washington University School of Medicine animal care facility. This facility is accredited by the Association for Assessment and Accreditation of Laboratory Animal Care (AALAC) and conforms to the PHS guidelines for Animal Care. Accreditation - 7/18/97, USDA Accreditation: Registration # 43-R-008.

## Author Contributions

P-YD, OA, VC, and VK conceived and designed the experiments and wrote the manuscript. P-YD and OA performed the experiments and data analysis. VC and VK secured the funding. All authors contributed to the article and approved the submitted version.

## Conflict of Interest

The authors declare that the research was conducted in the absence of any commercial or financial relationships that could be construed as a potential conflict of interest.

## Publisher’s Note

All claims expressed in this article are solely those of the authors and do not necessarily represent those of their affiliated organizations, or those of the publisher, the editors and the reviewers. Any product that may be evaluated in this article, or claim that may be made by its manufacturer, is not guaranteed or endorsed by the publisher.
