# Supplementary figures and images for: Hyperexcitability of Sensory Neurons in Fragile X Mouse Model
Source: Front Mol Neurosci. 2021 Dec 22;14:796053. doi: 10.3389/fnmol.2021.796053 (PMC8727524; doi:10.3389/fnmol.2021.796053)

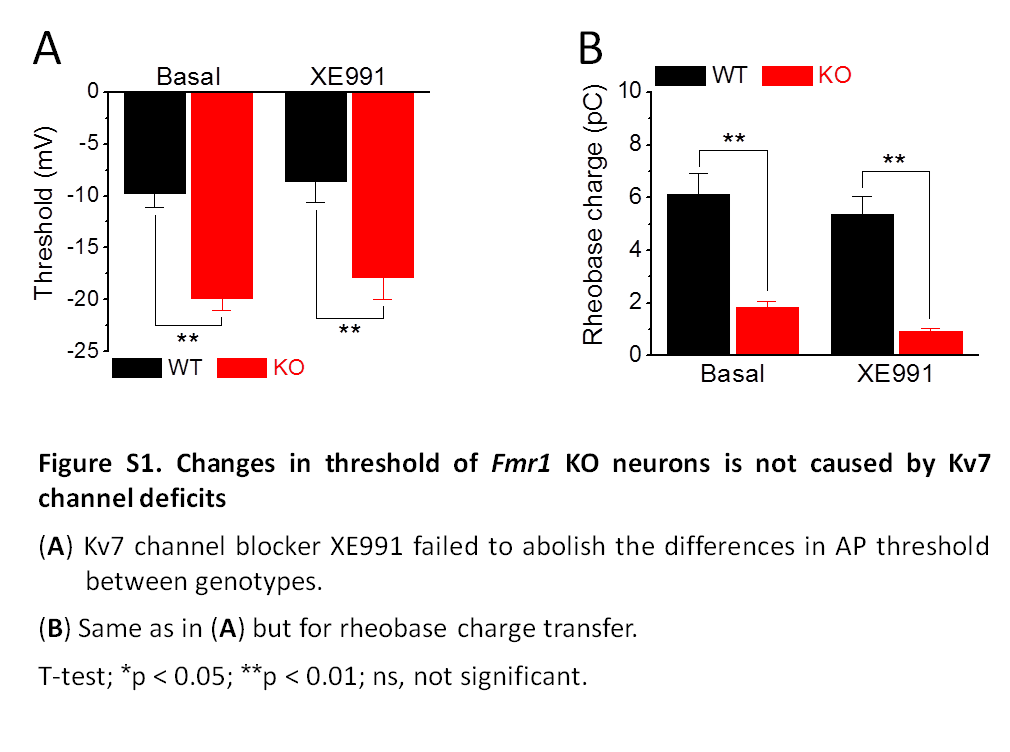

Supplement: Supplementary file 2 [file Image_1.tif]
